# Early Standard Electroencephalogram Abnormalities Predict Mortality in Septic Intensive Care Unit Patients

**DOI:** 10.1371/journal.pone.0139969

**Published:** 2015-10-08

**Authors:** Eric Azabou, Eric Magalhaes, Antoine Braconnier, Lyria Yahiaoui, Guy Moneger, Nicholas Heming, Djillali Annane, Jean Mantz, Fabrice Chrétien, Marie-Christine Durand, Frédéric Lofaso, Raphael Porcher, Tarek Sharshar, Groupe d’Explorations Neurologiques en Réanimation (GENER)

**Affiliations:** 1 Department of Physiology – Assistance Publique Hôpitaux de Paris, Raymond Poincaré Hospital, U1179 INSERM, University of Versailles Saint-Quentin en Yvelines - Garches, France; 2 General Intensive Care Medicine – Assistance Publique Hôpitaux de Paris, Raymond Poincaré Hospital, U1173 INSERM, University of Versailles Saint-Quentin en Yvelines - Garches, France; 3 Department of Anesthesiology and Intensive Care Medicine – European Hospital Georges Pompidou, Paris Descartes University, Paris, France; 4 Laboratory of Human Histopathology and Animal Models – Institut Pasteur – Paris, France; 5 Department of Statistics - Assistance Publique Hôpitaux de Paris, Hotel Dieu Hospital, University of René Descartes (Paris V) - Paris, France; University of Toronto, CANADA

## Abstract

**Introduction:**

Sepsis is associated with increased mortality, delirium and long-term cognitive impairment in intensive care unit (ICU) patients. Electroencephalogram (EEG) abnormalities occurring at the acute stage of sepsis may correlate with severity of brain dysfunction. Predictive value of early standard EEG abnormalities for mortality in ICU septic patients remains to be assessed.

**Methods:**

In this prospective, single center, observational study, standard EEG was performed, analyzed and classified according to both Synek and Young EEG scales, in consecutive patients acutely admitted in ICU for sepsis. Delirium, coma and the level of sedation were assessed at the time of EEG recording; and duration of sedation, occurrence of in-ICU delirium or death were assessed during follow-up. Adjusted analyses were carried out using multiple logistic regression.

**Results:**

One hundred ten patients were included, mean age 63.8 (±18.1) years, median SAPS-II score 38 (29–55). At the time of EEG recording, 46 patients (42%) were sedated and 22 (20%) suffered from delirium. Overall, 54 patients (49%) developed delirium, of which 32 (29%) in the days after EEG recording. 23 (21%) patients died in the ICU. Absence of EEG reactivity was observed in 27 patients (25%), periodic discharges (PDs) in 21 (19%) and electrographic seizures (ESZ) in 17 (15%). ICU mortality was independently associated with a delta-predominant background (OR: 3.36; 95% CI [1.08 to 10.4]), absence of EEG reactivity (OR: 4.44; 95% CI [1.37–14.3], PDs (OR: 3.24; 95% CI [1.03 to 10.2]), Synek grade ≥ 3 (OR: 5.35; 95% CI [1.66–17.2]) and Young grade > 1 (OR: 3.44; 95% CI [1.09–10.8]) after adjustment to Simplified Acute Physiology Score (SAPS-II) at admission and level of sedation. Delirium at the time of EEG was associated with ESZ in non-sedated patients (32% vs 10%, p = 0.037); with Synek grade ≥ 3 (36% vs 7%, p< 0.05) and Young grade > 1 (36% vs 17%, p< 0.001). Occurrence of delirium in the days after EEG was associated with a delta-predominant background (48% vs 15%, p = 0.001); absence of reactivity (39% vs 10%, p = 0.003), Synek grade ≥ 3 (42% vs 17%, p = 0.001) and Young grade >1 (58% vs 17%, p = 0.0001).

**Conclusions:**

In this prospective cohort of 110 septic ICU patients, early standard EEG was significantly disturbed. Absence of EEG reactivity, a delta-predominant background, PDs, Synek grade ≥ 3 and Young grade > 1 at day 1 to 3 following admission were independent predictors of ICU mortality and were associated with occurence of delirium. ESZ and PDs, found in about 20% of our patients. Their prevalence could have been higher, with a still higher predictive value, if they had been diagnosed more thoroughly using continuous EEG.

## Introduction

Sepsis is frequently complicated by brain dysfunction, ranging from delirium to coma and is associated with increased mortality and long-term cognitive dysfunction [1,2,3,4,5]. Brain dysfunction is related to both neuro-inflammatory and ischemic processes resulting in neuronal dysfunction [1,5,6]. EEG abnormalities observed in septic patients may reflect these neurotoxic processes [4,7,8,9,10]. Evoked potentials studies revealed impairment of brain function during severe sepsis [11]. Characteristic EEG patterns during sepsis mainly include lack of reactivity, excessive theta and delta activity, electrographic seizures (ESZ) and periodic discharges (PDs) [7]. The prevalence of these changes vary according to study design (i.e. prospective versus retrospective), the studied population (i.e. medical ICU versus surgical ICU), the severity and the time course (i.e. acute versus post-acute phase) of sepsis but also according to the methods of recording (i.e. standard EEG versus continuous EEG). In a prospective cohort of 62 septic patients, Young et al. [10] reported that continuous generalized PDs with triphasic morphology (triphasic waves) and EEG suppressions correlated with the severity of brain dysfunction and with mortality. Recently, ESZ and PDs were shown to be associated with a poor outcome in two retrospective cohorts of critically ill patients with a high prevalence of patients suffering from sepsis, who underwent continuous EEG [7,8]. In a more recent study using continuous EEG in ICU septic patients, Gilmore and colleagues demonstrated that lack of EEG reactivity was associated with mortality up to 1 year after discharge. Non convulsive seizures and PDs were common in patients with severe sepsis and altered mental status, but were not associated with outcome in their study [12]. Continuous EEG is potentially useful, but its routine use remains less spread that standard EEG [13]. Delirium is associated with increased mortality and long-term cognitive dysfunction [14], yet a link between EEG abnormalities and subsequent occurrence of delirium has never been established in septic patients. Demonstrating such a relationship would enable us to use EEG-based strategies to prevent delirium and its consequences in this population. Finally, the prognostic value of an EEG recorded early after admission to the ICU for sepsis remains to be assessed.

The primary goal of this study was to assess whether early EEG changes in patients admitted to the ICU for sepsis could predict ICU mortality. The secondary goal was to assess the relationship between early EEG abnormalities and the occurrence of delirium.

## Materials and Methods

### Patients

This prospective observational study was conducted according to the Strengthening the Reporting of Observational Studies in Epidemiology (STROBE) guidelines [15], in the 15 bed mixed medical/surgical intensive care unit of a teaching hospital (Raymond Poincaré hospital, Garches, France), between November 2011 and April 2014. Authorization of our local ethics committee "Ethics Committee Groupement Hospitalier Universitaire (GHU) Paris Nord, Comité d’Ethique de Recherche Biomédicale" was obtained (number: CERB 11–071) prior to conducting this study. The study protocol was approved by the local institutional review board, and written informed consent was obtained from the closest relatives for all the studied patients. Patients were eligible for the study if admitted to the ICU for sepsis, defined according to international criteria [16]. Patients were excluded if they presented any of the following conditions: cerebral infection, cardiac arrest, clinical evidence of brain death, effective decision to withhold or withdraw active treatments, body temperature below 35°C, refusal by the patient or his legal representative to participate in the study.

### Baseline biological and clinical data collection

The following demographic data were collected at the time of EEG recording: age, gender, prior functional status assessed by the Knaus score [17], medium-term prognosis before acute disease assessed by the McCabe score [18] and date of admission to the ICU. Medical data such as medical history, initial severity (assessed by the SAPS-II score) [19], number and severity of organ failure at admission (assessed by the SOFA: Sepsis-related Organ Failure Assessment score) [20], source of infection and bacteriological documentation were collected. Biological data including: serum sodium, potassium, calcium, bilirubin, blood urea nitrogen, creatinine, arterial lactate, arterial pH, alkaline reserve, PaO2 and PaCO2 were also collected. Sepsis was defined as the presence of infection, probable or documented, together with systemic manifestations of infection [16,21]. Severe sepsis was defined as sepsis plus sepsis-induced organ dysfunction or tissue hypoperfusion with sepsis-induced tissue hypoperfusion defined as infection-induced hypotension, elevated lactate, or oliguria [16,21]. Septic shock was defined as sepsis-induced hypotension persisting despite adequate fluid resuscitation [16,21]. Sepsis-induced hypotension was defined as a systolic blood pressure < 90 mmHg or mean arterial pressure < 70 mmHg or a systolic blood pressure decrease > 40 mmHg or less than two standard deviations below normal for age in the absence of other causes of hypotension. [16,21]. A standardized neurological examination was performed at inclusion and every 12h. Every 6h, consciousness and alertness were assessed in non-sedated patients by means of the Glasgow Coma Score (GCS) and occurrence of delirium was defined and assessed by means of the confusion assessment method for the intensive care unit (CAM- ICU) scale [22]. In sedated patients, the type of administered drug was recorded and depth of sedation was assessed every 6h using the Richmond Agitation Sedation Scale (RASS) [23]. Patients were considered comatose if the GCS was less than or equal to 8 for non- sedated patients, or if the RASS was less than or equal to—4 in sedated patients.

### EEG Recordings and analysis

EEG was recorded, within 72 hours of admission to the ICU. Standard EEG was performed at the bedside, using a Nihon Kohden manufactured EEG-9100J/K portable digital EEG system. Methodology for EEG recordings and analysis followed guidelines of the International Federation of Clinical Neurophysiology for the use of EEG in the ICU [24,25]. Eleven electrodes were placed according to the international 10–20 system. EEG recordings lasted at 20 minutes and included reactivity testing. Standardized EEG analysis included a description of basic parameters: EEG reactivity to noise and painful stimuli, continuity, predominant frequency (alpha, beta, theta or delta), and voltage. EEG voltage was measured from peak to trough, in longitudinal bipolar montage with standard 10–20 electrodes (normal voltage: ≥ 20μV, low voltage: < 20 μV). EEG reactivity was tested by strong sound stimuli (slapping hands, calling aloud the patient's first name) and intense nociceptive stimuli (strongly pinch the patient's arm, his sternum and nipple). EEG reactivity involves change in amplitude or frequency following stimulation. However, EEGs with only appearance of muscle activity, eye blink artifacts or SIRPIDs in response to stimulation, were looked at separately from standard reactivity and were not qualify as reactive. Occurrence of ESZ and PDs were noted. Any rhythmic discharge or spike and wave pattern with definite evolution in frequency, location, or morphology, lasting more than 10 seconds was considered as an ESZ [26]. PDs were repetitive sharp waves, spikes, or sharply contoured waves at regular or nearly regular intervals and without clear evolution in frequency or location. PDs included generalized periodic discharges, lateralized periodic discharges, continuous generalized PDs with triphasic morphology (triphasic waves) and stimulus induced periodic, rhythmic and ictal appearing discharges [27]. EEG recordings were subsequently categorized using the Synek and the Young classifications [10,28]. The Young classification in septic encephalopathy ranges from 0 to 4, with grade 0: normal EEG; grade 1: predominant theta activity; grade 2: predominant delta activity; grade 3: predominant triphasic waves; and grade 4: suppression. The EEG classification developed by Synek includes: grade 0: normal EEG; grade 1: predominant alpha activity with or without some delta-theta activity; grade 2: predominant theta or delta activity with some alpha activity still detectable; grade 3: predominant theta or delta activity, no alpha activity; grade 4: low-voltage delta activity, burst suppression, alpha coma, theta coma, PDs, epileptiform paroxysms, a massive low-voltage background activity; and grade 5: electro-cerebral silence. Each EEG was independently analyzed offline by two senior neurophysiologists (EA and MCD) who were blinded to clinical data. We assessed inter-observer rate of concordance for the studied EEG parameters using the kappa analysis. Any disagreements were resolved by consensus reading.

### Follow up

Data were collected until day 28 of hospitalization or until ICU discharge. The following items were recorded: duration of mechanical ventilation, duration of administration of sedative drugs, time to awakening after sedation had been stopped defined by spontaneous eye opening and eye tracking, occurrence of delirium, using the CAM-ICU score and its duration, length of hospital and ICU stay and when applicable, date of death.

### Statistical analysis

Categorical variables were expressed as numbers and percentages and compared by Fisher's exact test. Quantitative variables were presented as mean standard deviation (SD) or median interquartile range [IQR] and compared using the Mann-Whitney test. Adjusted analyses were carried out using multiple logistic regression. Given the number of non-survivors in the ICU, we did not use model selection procedures with backward variable elimination, but simply performed analyses adjusted on the SAPS-II score at admission and the use of sedation and SOFA score at inclusion. Associations were considered statistically significant when the p-value was less than or equal to 0.05. Statistical analyses were performed using the R software version 3.0.1.

## Results

### Characteristics of the patients

**Fig 1:**
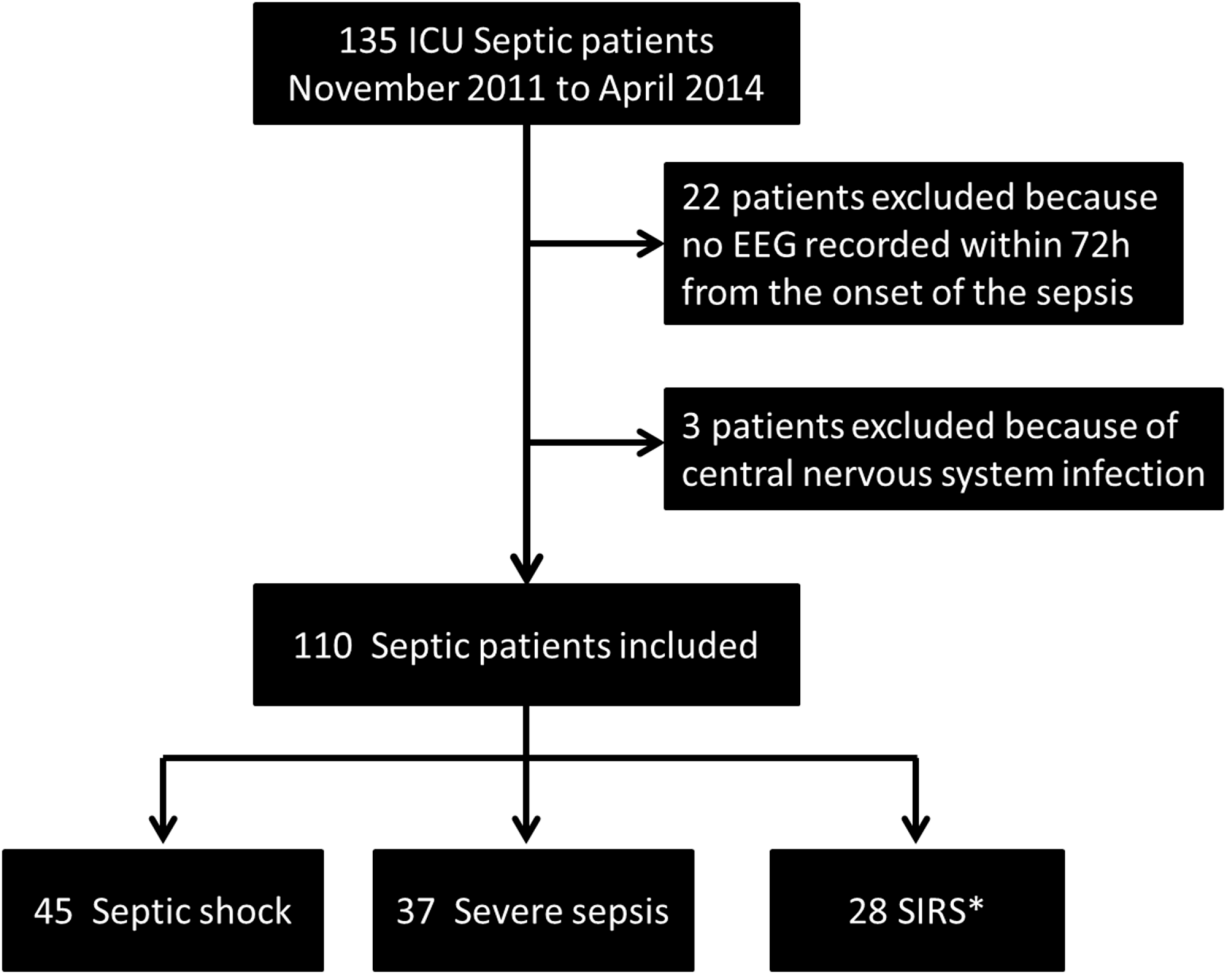
Flow chart of the recruited population. *: Systemic Inflammatory Response Syndrome.

From November 2011 to April 2014, 135 patients were admitted to our unit for sepsis. Twenty two patients were excluded from the study because their EEG was not performed within 72h of admission to the ICU for sepsis. Three patients were excluded because of central nervous system infection. Fig 1 shows a flow chart of the recruitement of the studied population. Overall, 110 patients were included. Median time from admission to the ICU to EEG recording was 1 [1–2] day. Patients’ characteristics are summarized in Table 1. Mean age was 63.8 (±18.1) years. Median SAPS-II and SOFA scores at admission were respectively 38 [29–55] and 6 [3–9]. Sixty five (59%) patients were mechanically ventilated, 45 (41%) suffered from septic shock. Sepsis was related to pneumonia in 74 (67%) of cases. Five (5%) patients had a previous history of epilepsy.

**Table 1:** Main characteristics at admission, inclusion and after hospital discharge.

Variables	
**At ICU admission**	
Number of included patients	110
Women—n (%)	32 (29)
Age (years)—mean (SD)	63.8 (18.1)
Epilepsy—n (%)	5 (5)
Alcoholism—n (%)	10 (9)
Site of infection—n (%)	
Lung	74 (67)
Abdominal	12 (11)
Urinary tract	12 (11)
Skin	7 (6)
Vascular	4 (4)
Other	1 (1)
Pathogens—n (%)	
Gram negative	23 (21)
Gram positive	15 (14)
Polymicrobial	15 (14)
Other	7 (6)
None	50 (45)
SAPS II at admission—median [IQR]	38 [29 to 55]
Knaus at admission—n (%)	
A	7 (6)
B	52 (47)
C	38 (35)
D	13 (12)
McCabe at admission—n (%)	
1	73 (66)
2	23 (21)
3	14 (13)
Time from admission to EEG (days)–median [IQR]	1 [1 to 2]
Time from onset of sepsis to EEG (days)–median [IQR]	1 [1 to 3]
**At the time of EEG recording**	
Severity of sepsis—n (%)	
SIRS	28 (25)
Severe sepsis	37 (34)
Septic shock	45 (41)
Neurological status—n (%)	
Delirium	22 (20)
No Delirium	42 (38)
Sedated	46 (42)
Type of sedation	
Midazolam—n (%)	41 (37)
Sufentanyl—n (%)	44 (40)
Propofol—n (%)	6 (5)
RASS*	2 [1 to 5]
SOFA (from 0 to 24)–median [IQR]	6 [3 to 9]
Renal SOFA (from 0 to 4)–median [IQR]	1 [1 to 2]
Liver SOFA (From to 4)–median [IQR]	0 [0 to 0]
Mechanical ventilation at time of EEG—n (%)	61 (55)
**After EEG recording**	
Sedated at time of EEG or after—n (%)	48 (44)
Duration of sedation (days)–median [IQR]	2 [1 to 5]
Mechanical ventilation at time of EEG or after—n (%)	65 (59)
Duration of mechanical ventilation (days)–median [IQR]	8 [4 to 23]
Delirium in patients with no delirium at inclusion—n (%)	32 (36)
In-hospital mortality—n (%)	23 (21)
Length of stay in the ICU (days)–median [IQR]	8 [3 to 18]

*Abbreviations*: *n*: *number*, SAPSII: Simplified Acute Physiology Score; EEG: electroencephalogram; SIRS: Systemic Inflammatory Response Syndrome; RASS: Richmond Assessment Sedation Scale; SOFA: Sepsis-related Organ Failure Assessment; ICU: Intensive Care Unit;

*Only in sedated patients. Delirium was defined according to the CAM-ICU.

### EEG findings and their relationship with severity of sepsis, sedation and neurological status

**Fig 2:**
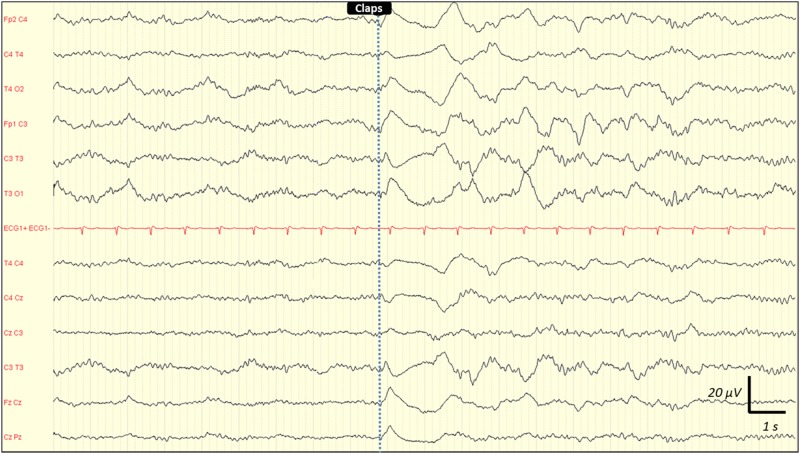
Representative example of a reactive EEG following auditory stimulation (claps) in a septic ICU patient. See the diffuse and synchronic slowing of the EEG background activity, that appeared immediately after the auditory stimulation (claps). Recording: 20 mm/sec, sensitivity: 10 μV/mm; filter settings: 0.500 Hz -70Hz.

**Fig 3:**
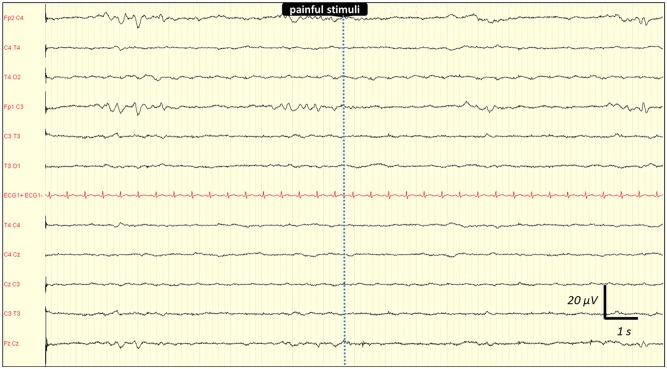
Representative example of a non-reactive EEG following painful stimuli (pinching) in a septic ICU patient. No EEG background activity change was observed after the painful stimulation (pinching) Recording: 20 mm/sec, sensitivity: 10 μV/mm; filter settings: 0.500 Hz -70Hz.

**Fig 4:**
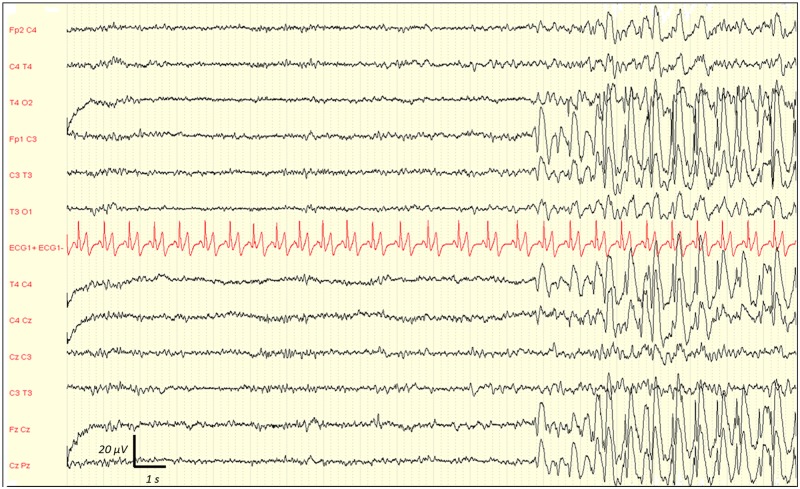
Representative example of EEG showing the onset of an electrographic seizure (ESZ) in a septic ICU patient. See the onset of this spontaneous and sustained discharge of spikes and waves with changes in frequency and morphology. Recording: 20 mm/sec, sensitivity: 10 μV/mm; filter settings: 0.500 Hz -70Hz.

**Fig 5:**
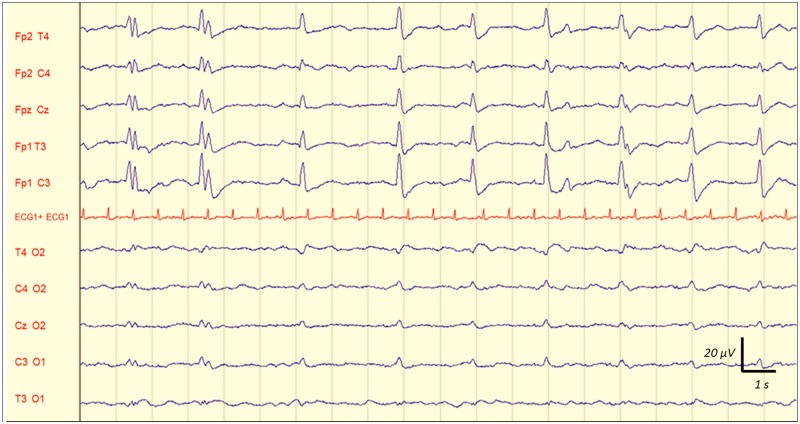
Representative example of EEG showing periodic discharges (PDs) in a septic ICU patient. See these spontaneous generalized repetitive discharges of spikes and bursts of sharp waves at nearly regular intervals and without evolution in location and morphology. Recording: 20 mm/sec, sensitivity: 10 μV/mm; filter settings: 0.500 Hz -70Hz.

**Fig 6:**
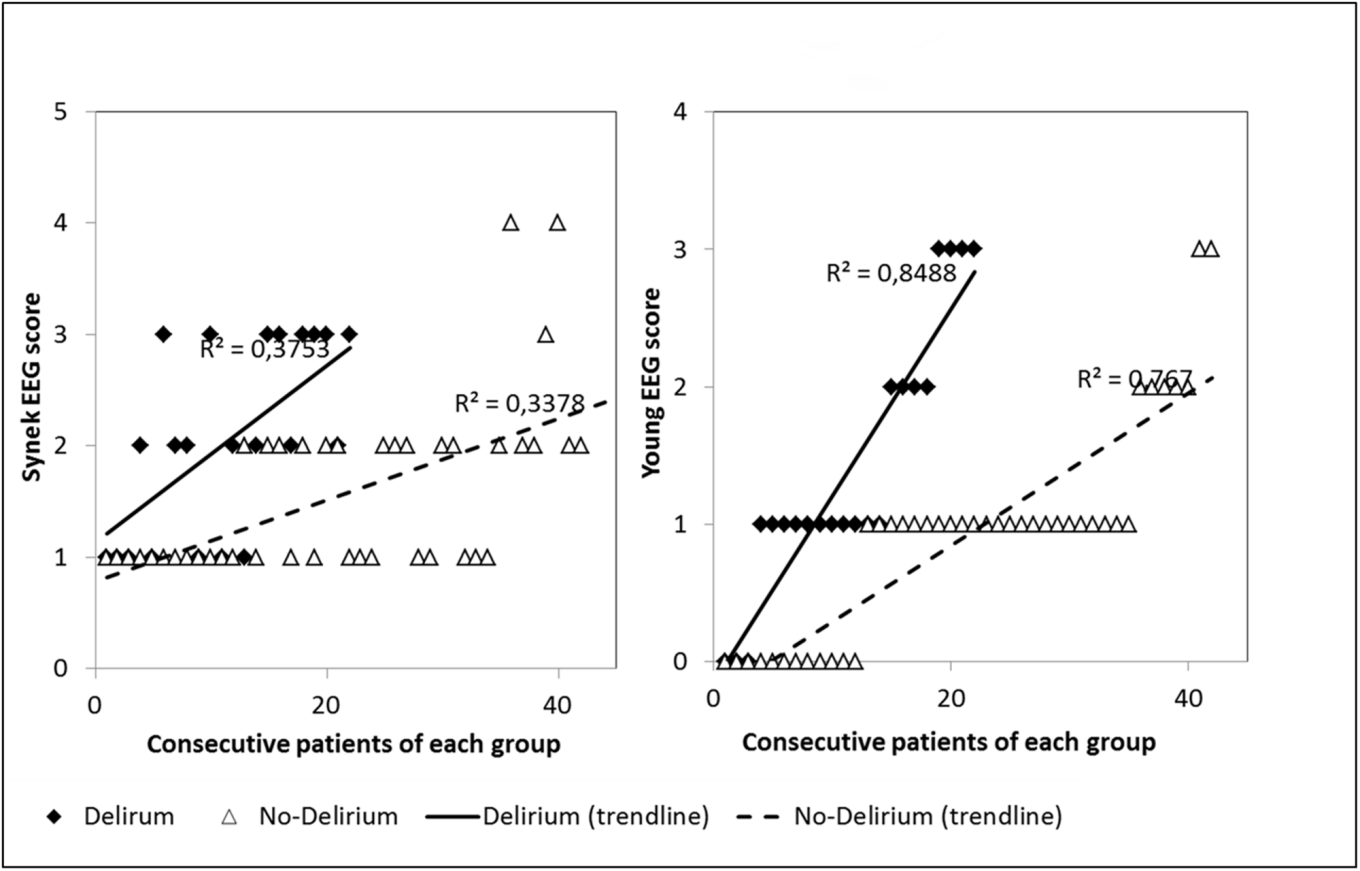
Scatter plots of Synek and Young EEG scales in the Delirium versus No-Delirium groups.

**Fig 7:**
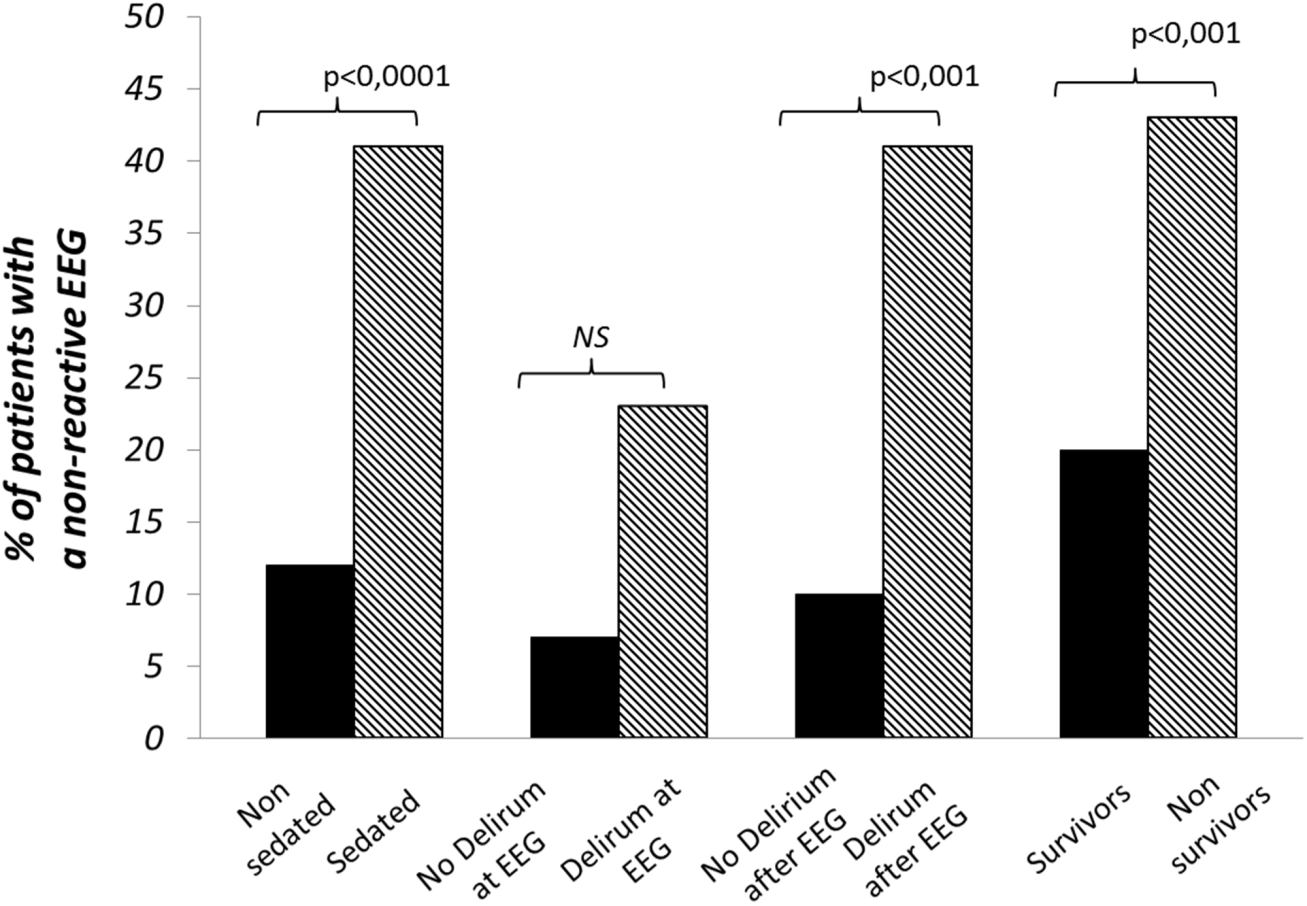
Prevalence of non-reactive EEG among different sub-groups of the studied septic ICU patients.

Inter-observer agreement for interpreting EEG findings was excellent. Observed agreement was 100% (kappa = 1) for absence of reactivity, triphasic wave, ESZ and PDs. It was 99% (kappa = 0.98) for amplitude and Young's classification, 97% (kappa = 0.96) for the dominant frequency, and 95% (kappa = 0.92) for the Synek's classification. Any disagreements were resolved by consensus reading. EEG rhythms were most frequently in the theta frequency band n = 53 (48%) (Table 2). ESZ were observed in 17 (15%) cases, PDs in 21 (19%) and absence of reactivity in 27 (25%) cases. Among the 21 patients with PDs, 7 had only PDs without any ESZ. Among the 17 patients with ESZ, 14 had both ESZ and PDs and 3 patients had ESZ without PDs. This shows that most patients with ESZ had also PDs: 14/17 (82%). Fig 2 is a representative example of a reactive EEG after auditory stimulation (claps), and the Fig 3 is a representative example of a non-reactive EEG after intense painful stimuli (pinching). Fig 4 shows the example of onset of an ESZ and Fig 5 is an example of EEG featuring PDs. Forty six patients (42%) were sedated at the time of EEG, median RASS score was 2 [1–5]. Median length of sedation was 2 [1–5] days. Patients with septic shock were more frequently sedated (Table 3). Sedation was significantly associated with delta activity (p = 0.0004), absent reactivity (p = 0.0007), decreased amplitude (p < 0.0001), Synek Grade ≥ 3 (p = 0.0003) and Young Grade > 1 (p = 0.0006). ESZ (p = 0.60) and PDs (p = 0.63) were less frequent—yet not significantly—in sedated patients. EEG patterns did not correlate with age, time since onset of sepsis, SAPS-II score, SOFA score and liver or kidney SOFA sub-scores. The infusion rate of midazolam did not differ between patients with and without EEG reactivity (4.5 ± 2.5 mg/h versus 5.9 ± 4.5 mg/h, p = 0.21. Twenty two (20%) patients suffered from delirium at the time of EEG recording and 32 (36%) patients subsequently developped delirium. Delirium could not be assessed in eight patients. The overall prevalence of delirium was 50%. In non-sedated patients delirium at the time of EEG was associated with ESZ (7 (32) vs 4 (10); p = 0.037), Synek score (p = 0.02) and Young score (p = 0.048), (Table 3). The Fig 6 shows scatterplots of Young and Synek EEG scales in the delirium versus no delirium groups at the time of EEG recording. Occurrence of delirium in the days after EEG recording was associated with the SAPS-II score (p = 0.001), septic shock (p = 0.003), delta frequency dominant EEG (p = 0.001), absence of EEG reactivity (p = 0.003), Synek grade ≥ 3 (p = 0.021) and Young grade ≥ 1 (p = 0.0001) (Table 3). Fig 7 shows the prevalence of absent EEG reactivity among different sub-groups of outcome.

**Table 2:** EEG findings and classification.

Main EEG findings	
Total number of EEG	110
Dominant frequency—n (%)	
alpha	21 (19)
delta	36 (33)
theta	53 (48)
Amplitude—n (%)	
low-voltage	71 (65)
normal	39 (35)
Absence of reactivity—n (%)	27 (25)
Electrographic seizure—n (%)	17 (15)
Periodic Discharges—n (%)	21 (19)
Triphasic waves—n (%)	7 (6)
Synek’s scale (from 0 to 5)–median [IQR]	2 [1 to 3]
Synek’s score ≥ 3 –n (%)	34 (31)
Young’s scale (from 0 to 4)–median [IQR]	1 [1 to 2]
Young’s score > 1 –n (%)	41 (37)

**Table 3:** Comparisons of clinical data and EEG findings between different sub-groups of patients: sedated versus non-sedated at time of EEG, with versus without delirium at time of EEG, with versus without delirium at follow up (after EEG) and survivors versus non survivors.

Variables		Sedated at time of EEG recording		Delirium at time of EEG recording (£)		Developed Delirium after EEG (¥)		In hospital Death	
		yes	no	yes	no	yes	no	yes	no
Number of patients	n (%)	46 (42)	64 (58)	22 (20)	42 (38)	32 (29)	48 (44)	23 (21)	87 (79)
Age (years)	mean (SD)	62.8 (16.0)	64.5 (19.6)	66.1 (19.8)	63.6 (19.6)	66.4 (16.9)	61.3 (18.9)	66.1 (15.9)	63.3 (18.5)
SAPS-II at admission	median [IQR]	54 ***	34	42 *	33[25 to 40]	45 **	34	44	37
		[37 to 66]	[27 to 45]	[30 to 53]	[25 to 40]	[36 to 63]	[27 to 47]	[36 to 65]	[28 to 54]
Septic shock	n (%)	30 (65) ***	15 (23)	8 (36)	7 (17)	20 (62)**	13 (27)	9 (39)	36 (41)
SOFA at inclusion	median [IQR]	9 ***	3	4 **	3	7 ***	4	10 **	5
		[7 to 12]	[2 to 5]	[3 to 7]	[2 to 4]	[5 to 11]	[2 to 7]	[6 to 15]	[3 to 7]
**EEG findings**									
Delta-predominant	n (%)	24 (52)***	12 (19)	7 (32)	5 (12)	16 (50)**	7 (15)	11 (48)*	25 (29)
Low voltage	n (%)	40 (87)***	31 (48)	11 (50)	20 (48)	25 (78)	28 (58)	14 (61)	57 (65)
Absence of reactivity	n (%)	19 (41)***	8 (12)	5 (23)	3 (7)	13 (41) **	5 (10)	10 (43)**	17 (20)
Electrographic seizure	n (%)	6 (13)	11 (17)	7 (32)*	4 (10)	5 (15)	5 (10)	4 (17)	13 (15)
Triphasic waves	n (%)	0 (0) *	7 (11)	4 (18)	3 (7)	1 (3)	2 (4)	2 (9)	5 (6)
Periodic Discharges	n (%)	10 (22)	11 (17)	5 (23)	6 (14)	8 (25) *	5 (10)	7 (30)*	14 (16)
Synek score ≥ 3	n (%)	23 (50)***	11 (17)	8 (36)**	3 (7)	14 (44)**	8 (17)	12 (52)***	22 (25)
Young score > 1	n (%)	26 (57)***	15 (23)	8 (36)	7 (17)	19 (59)***	8 (17)	12 (52)**	29 (33)

^£^: Delirum could not be assessed at the time of EEG recording in the 46 patients because they were sedated.

^¥^: The 22 patients with delirium at time of EEG (inclusion) were not included in this analysis, and delirium could not be assessed in 8 patients. SAPSII: New Simplified Acute Physiology Score; SOFA: Sepsis-related Organ Failure Assessment. p values were from univariate analysis. Difference was considered significant if p < 0.05:

* = p < 0.05;

** = p < 0.001;

*** = p < 0.0001.

### Relationship between EEG abnormalities and mortality

Twenty three deaths (21%) occurred during the ICU stay (Table 1). Causes of death were refractory hypotension (n = 5), refractory hypoxemia (n = 6), multiple organ failure (n = 10), myocardial infarction (n = 1) and ventricular arrhythmia (n = 1). Decisions to withhold or to withdraw care were made for seven patients. On univariate analysis, mortality did not correlate with age, SAPS-II score, delirium or severity of sepsis but did correlate with the SOFA score at inclusion (p = 0.001), absence of EEG reactivity (p = 0.002), occurrence of PEDs (p = 0.043), Synek Grade ≥ 3 (p = 0.0007) and Young Grade > 1 (p = 0.007) (Table 3). Multivariate analysis revealed that ICU mortality was independently associated with absence of EEG reactivity, Synek Grade ≥ 3 and Young Grade > 1 when adjusted to SAPS-II score at admission and sedation as well as when adjusted to SOFA at inclusion and sedation (Table 4).

**Table 4:** Adjusted analysis of the association of EEG findings with day 28 mortality.

Variable	Adjusted on SAPS-II at admission and sedation			Adjusted on SOFA at EEG and sedation		
	OR	(95%CI)	P	OR	(95%CI)	P
Delta-dominant activity	3.36	(1.08 to 10.4)	0.036	3.08	(0.93 to 10.2)	0.066
Absence of reactivity	4.44	(1.37 to 14.3)	0.013	4.57	(1.36 to 15.4)	0.014
Periodic Discharges	3.24	(1.03 to 10.2)	0.044	3.31	(0.98 to 11.2)	0.054
Synek’s score ≥ 3	5.35	(1.66 to 17.2)	0.005	5.68	(1.63 to 19.8)	0.006
Young’s score > 1	3.44	(1.09 to 10.8)	0.035	3.43	(1.02 to 11.6)	0.046

*Abbreviations*: SAPS-II: New Simplified Acute Physiology Score; SOFA: Sepsis-related Organ Failure Assessment.

## Discussion

In this prospective observational cohort study using early standard short duration EEG (20 minutes) in 110 patients with sepsis of any severity, we found that: 1) delirium at the time of EEG correlated with Young and Synek scale scores and seizures in non-sedated patients; 2) later development of delirium correlated with a delta-predominant background, Young and Synek scale scores, and absence of reactivity; and 3) mortality correlated with delta background, absence of reactivity, Young and Synek scores. Obtained in a population of septic patients with 20% mortality rate and a prevalence of delirium of 50%, these findings indicate that early standard EEG is helpful for predicting mortality and identifying patients at risk to develop delirium.

Few studies have assessed EEG abnormalities in septic patients, and prospective studies are scant. The type of sepsis related-EEG abnormalities, their prevalence and prognostic value vary among existing studies. Young et al [10] reported that continuous generalized PDs with triphasic morphology and suppression (including burst-suppressions) correlated with the severity of septic encephalopathy and with mortality. The absence of EEG reactivity did not differentiate survivors from non survivors. However EEG reactivity was only tested in the subgroup of patients suffering from severe encephalopathy. In a retrospective study of 201 patients from a medical ICU who underwent continuous EEG [8], sepsis was the only significant predictor of ESZ and PDs. ESZ and PDs were both associated with death or severe disability at hospital discharge. These findings were partially confirmed by the same team in another retrospective cohort of surgical ICU patients undergoing continuous EEG [7], where ESZ were associated with poor outcome. Our study, along with that of Young et al [10] indicates that standard EEG enables the detection of sepsis-related EEG changes. We found that 14/17 (82%) patients with ESZ had also PDs. This co-occurrence of ESZ with PDs is also in accordance with other recent studies involving ICU septic patients, suggesting that sepsis was a significant risk factor for EZS and PDs [7,8]. The co-occurrence of ESZ and PDs in septic ICU patients may then be seen as a potential marker of brain dysfunction with subsequent prognostic significance in this specific population of patients. Regarding the link between lack of EEG reactivity and mortality, our findings were in accordance with the ones of the recent study by Gilmore and colleagues using continous EEG [12]. But ESZ and PDs were not associated with death in their study. However, it should be mentioned that their study was about a much sicker population, all with impaired mental status (required to get EEG), and with a much higher mortality. The main originality of the present study lies in the fact that EEG was recorded early after admission to the ICU and that delirium was systematically assessed during follow up, enabling us to identify early predictors of both brain dysfunction and of death.

We assessed EEG reactivity after standardized noise and noxious stimuli [24,25]. EEG reactivity requires the integrity of the peripheral sensory or auditory pathways, as well as integrity of the brainstem, sub-cortical structures and cerebral cortex. Absent reactivity can result from the dysfunction of any one of these structures, including the brainstem. Sedation was managed following international recommendations. Indeed median RASS and duration of sedation were respectively– 2 and 2 days. Absence of EEG reactivity did not correlate with midazolam infusion rates or with the RASS score. Absence of EEG reactivity was therefore not related to excessive sedation. This was expected as the neurological effect of midazolam is liable to inter-individual variability [29]. To better assess the relationship between sedation and EEG features, it would have been necessary to adjust sedation to neurophysiological features, or at least to repeat EEGs during the course of sedation. Interestingly, Shehabi et al [30] reported that a RASS below -3 was independently associated with increased mortality in critically ill patients. Absence of EEG reactivity might have been a confounder; unfortunately EEG recordings were not performed. The relationship between the absence of EEG reactivity and subsequent occurrence of delirium is intriguing. This relationship might be related to the administration of midazolam, which has been shown to increase the risk of delirium [14]. Determining if preserving reactivity EEG reactivity at an early phase of sedation reduces both mortality rates and the prevalence of delirium would warrant a clinical trial. A better understanding of the mechanisms of EEG unresponsiveness would empower the development of such a specific therapeutic approach. Additionally, comparisons with other types of encephalopathy in which lack of EEG reactivity have been shown to be of prognostic value (such as post-anoxic, post-traumatic or hepatic encephalopathy), could be useful [31,32,33].

A recent study showed that burst suppression were associated with subsequent occurrence of delirium in sedated critically ill patients [34]. We found a similar relationship between delirium and Young grade > 1 and Synek grade ≥ 3, which both include burst suppression. In our study three patients had EEG with burst suppression. All were sedated at time of EEG recording, two developed delirium at follow-up and one died.

We found that PDs were associated with mortality, similarly to previous reports [8]. There is no obvious explanation for such a relationship. Firstly, the fact that EEGs were not repeated over time hampers the determination as to whether PDs are predictive of subsequent EEG deterioration, and the nature thereof. The use of continuous EEG (24 to 48 hours) would have probably allowed a better detection of ESZ and PDs and therefore a better assessment of their prognosis value [26]. Secondly, both the pathophysiology and the significance of PDs are a matter of debate. PDs have been demonstrated to be associated with a high risk of seizures [35,36,37].

Interestingly, we found an association between ESZ and delirium at the time of EEG recording in non-sedated patients. We specifically assessed the sub-population of non-sedated patients, as sedation might have masked or treated ESZ. Our findings suggest that an epileptic process is involved in sepsis-induced delirium. Epilepsy is a well-documented cause of confusion and sepsis is capable of inducing ESZ [8]. Epileptic activity may be secondary to impairment of cortico-subcortical loops, as these loops are necessary for arousal, consciousness and attention; functions that are preferentially altered in delirium [38,39,40]. Epileptic/epileptiform phenomena, along with neurotransmission imbalance, impairment of long-term potentiating or neuronal apoptosis are markers of neurotoxicity resulting in or caused by a neuro-inflammatory process [1,5,6]. This neuro-inflammatory process includes endothelial activation, blood-brain barrier and astrocyte dysfunction and microglial activation, resulting in the release of neurotoxic mediators which induces cellular stress (notably oxidative stress and mitochondrial dysfunction). Moreover, it is well known that, microglial activation increases central nervous system excitability and, reciprocally, epileptic processes induce neuro-inflammation [41]. We can hypothesize that the prevention or treatment of ESZ might be beneficial. Anti-epileptic drugs have never been tested in septic patients but have been shown to be useful and safe for preventing early seizures in patients with traumatic brain injury [42,43]. Emphasis must also be placed on prevention and treatment of ESZ by avoiding drug neurotoxicity (notably antibiotics), treating metabolic disturbances and preventing drug or alcohol withdrawal symptoms -especially of benzodiazepines and opioids [44].

Our study has several limitations:

- A small sample size, obtained from a single center cohort study. This weakness may have favored the selection of patients mainly with sepsis of mild severity. The relatively high survival rate also indicates that mainly patients with sepsis of mild severity have been enrolled. Thus, it is conceivable that the early EEG predictors identified in our study may not be immediately extrapolable to severe sepsis or septic shok.; conversely, it cannot be ruled out that, increasing the sample size may have strengthened the statistical associations obtained between EEG changes and outcome parameters.
- The inclusion of both sedated and nonsedated patients: We included sedated patients because on the one hand, severe patients tend to require sedation to ensure their confort and safety but are at a higherrisk of developing acute brain dysfunction; on the other hand, EEG recording remains informative to some extent despite sedation. Although sepsis is known to favor a decrease in drug clearance, ESZ and PDs cannot be caused by the administration of benzodiazepines or opioids, which were the most common agents used in our patients. It can be argud that we have not assessed the specific EEG features of sepsis-associated encephalopathy but rather those of a combination of sepsis and sedation, among others, sinces 42% of the patients were sedated during EEG recording.
- The lack of continuous EEG: In comparison to continuous EEG or recording of longer duration (30 to 60 minutes), we used EEG recording of short duration (20 minutes) which does not allow to provide an accurate prevalence of EEG changes in septic patients, notably of ESZ [26]. However, we would like to emphasize that our study was a pragmatic one, and that the conditions complied with the recommendations of EEG use for ICU patients in France [24,25,45]. Our results indicate that a 20 min EEG recording (which is the most frequent duration of EEG recording for ICU patients in France) is helpful for predicting the outcome of septic patients admitted in ICU. Continuous EEG enables a more accurate detection of EEG changes, but is not currently available in most of the ICUs in France. Alternatively, it is possible that repeated EEG recordings would have enabled us to refine neurophysiological predictors. Indeed, duration of EEG abnormalities or absence of EEG improvement are associated with poor outcome in various encephalopathies, including of septic origin [7]. Recently, Sutter and colleagues demonstrated a link between lack of electroencephalographic sleep elements (K-complexes, vertex sharp-waves and sleep spindles) and poor outcome in acute encephalopathic patients, especially in the elderly and patients suffering from septic shock [46].

## Conclusion

In this prospective cohort study of 110 septic ICU patients, we found that some early changes of standard EEG are associated with increased mortality and occurrence of delirium. These results suggest that strategies based on EEG might be useful for improving outcomes of septic patients. We would like to emphasize, that even if some EEG patterns are associated with mortality, EEG should not be used as a prognostic tool for making decisions regarding withdrawal of life support.
